# Michigan State Medical Society

**Published:** 1888-07

**Authors:** 


### THE MICHIGAN STATE MEDICAL SOCIETY.

TWENTY-THIRD ANNUAL MEETING AT DETROIT.

The address of welcome was delivered by John Pridgeon, mayor of the city.

Applications for membership were then read by the Secretary, and were all referred to the appropriate committee.

H. O. Walker, M. D., Chairman of the Executive Committee, reported the programme of the meeting, which was accepted.

Dr. George Duffield, Chairman of the Publication Committee, reported that 810 copies of the transactions of the last meeting were prepared, and 500 mailed, at an average cost of seven cents a volume. In consequence of the delay occasioned by the tardy receipt of papers, the committee recommended the adoption of the following:

*Resolved*, That papers not sent to the Publication Committee, complete, two weeks after adjournment shall not be included in the society's transactions.

On motion, all portions of the report referring to money matters were referred to the Finance Committee, except a provision for binding the report, which was laid on the table for one year. The rest of the report, including the resolution, was accepted and adopted.

W. J. McHensh, M. D.. Chairman of the Committee on Necrology, reported the deaths of Drs. A. B. Palmer and E. S. Dunster, of the University of Michigan, and Douglas A. Joy, of Marshall. He then read the memorial of Dr. Joy, and Dr Conrad George, of the University of Michigan, read the memorials of the two professors in the State University.

Dr. George Duffield, Secretary of the society, submitted his report, which commenced with a comparison of the status of the society at the last meeting held at Detroit (nine years ago) and at present. At the former meeting there was a membership of 168, of whom only seventy-five were present at the meeting, while now the membership is 275, of whom a very large proportion will be present; still the membership is small compared with the number of practitioners in the State, and earnest efforts have been made and are still making to induce members of the local societies to unite with the State organization.

A proposed amendment to the constitution, offered at a previous meeting, making all active members of the association removing from its jurisdiction eligible to corresponding membership, was put upon its passage and unanimously adopted.

H. B. Hemenway, M. D., Treasurer, reported as his gross receipts $920.26 and disbursements $848.32, leaving a balance of $71.94, but as $100 has been voted and not paid to the "Rush Memorial Fund" there is an actual deficit of $28.06.

The following was accepted us a substitute for sec. 3 article 3 of the by-laws:

"Membership dues shall be $2 per annum, and payable annually. The failure of any member to pay such dues for two successive years shall be reported by the Secretary of the society, and thereupon such delinquent shall be declared, by the presiding officer, suspended from all the rights and privileges of membership therein, except that of attendance upon its sessions; and a failure for five successive years to pay such dues, shall also be, in like manner reported, and the membership of such delinquent shall be declared forfeited and lost: Provided, that one so suspended may be restored to the full privileges of membership by a vote of the society upon the payment by him to the Secretary of the full amount of his unpaid dues.

"All applicants for membership who have forfeited their membership by the nonpayment of dues, shall be required to state in their application the fact of such forfeiture."

The President appointed the following nominating committee: Drs. Hitchcock, Kalamazoo; Hoyt West, Detroit; Breakey, Ann Arbor; Raynale, Brighton, and Langlois, of Wyandotte.

Dr. Wade, of Holly, made a humorous speech, protesting against the meetings of sections and against the reading of long papers that interested nobody. He did not come for instructions, but for pleasure, and he wished to see more of the social and less of the scientific element cultivated.

AFTERNOON SESSION.

In the Section on Practice of Medicine, of which A. W. Alvord, of Battle Creek, is chairman, the first paper on the programme, "Is Disease an Entity?" by Dr. C. H. Stowell, of Ann Arbor, was omitted on account of the absence of Dr. Stowell. The papers read and discussed were: "True Principles of House Drainage," Dr. S. P. Duffield; "Undrained Lands Predispose to Phthisis Pulmonalis," Dr. H. F. Lyster; "Tuberculosis, Etiology and Frequency," Dr. J. Flinterman; "The Pathology of Acute Miliary Tuberculosis," Dr Horace Gibbes.

The following papers were presented in the Section on Surgery, Dr. Donald Maclean, Detroit, chairman: "Retention of the Urine Relieved by Supra-pubic Paracentesis of the bladder," Dr. Charles F. Southworth; "A Demonstration in Abdominal Surgery," Dr. Hal. C. Wyman; "A Case of Splenectomy with Subsequent Liberation of the Ligature through the Air Passages," Dr. T. A. McGraw; "Some Eye Affections of the Veneral Diseases," Dr. Flemming Carrow, Bay City.

The Section on Obstetrics and Gynæcology, Dr. George E. Ranney, of Lansing, chairman, listened to and discussed the following papers: "An Address on Obstetrics and Gynæcology," Dr. G. E. Ranney; "Curability of Tumors of the Womb Without Severe Surgical Treatment," Dr. C. H. Leonard; "Puerperal Septicæmia," Dr. Charles H. Baker; "Uterine Flexions as a Cause of Anomalous Symptoms in Distant Organs," Dr. W. W. Elmer; "The Therapeutical use of the Pessary," Dr. H. J. Chadwick, and "Experimental Researches Respecting the Influence of Dress as a Cause of Pelvic Disorders in Women," Dr. J. H. Kellogg.

In the evening Dr. McGraw delivered an address at the opera house. One of the first sentences of his address, in speaking of the medical profession, was: "We are too much occupied with our own influence on people to pay sufficient attention to the influence of people on ourselves." He proceeded to show that it is impracticable, owing to national peculiarities, for foreign and more mature systems of medical education to be adopted in American schools. The English mind was too intensely practical; the German too philosophical. American medical and surgical schools are not put in a state of efficiency satisfactory to medical men. The responsibility of their low standard and the lack of equipment in hospitals should be assumed as much by the people as by the medical profession. He denounced the custom of permitting students to begin study in private offices under preceptors who seldom pay any attention to their needs and less to their fitness to practice. The excellence of the German schools was universally admitted, but the American system of training and education, though comparatively in its youth, produces in time results of which the profession and people may be proud. The American schools make their instruction eminently practical.

Dr. George K. Johnson, of Grand Rapids, followed with the annual address on the practice of medicine, his subject being "The Natural History of Disease the Basis of Rational and Scientific Practice."

He set forth that the vast majority of diseases end in return to health of those afflicted; that most sick people will get well when left to the curative influences of nature. He held that drug medication was too excessive and indiscriminate; that with few exceptions, the knowledge of drug action did not warrant prodigality in usage. The present drift was in the direction of conservatism in the use of drugs, although there was a constant flood of therapeutic novelties. The only course for the true physician was to take the law from science only.

The closing feature of the programme was the annual address on surgery by Dr. Geo. E. Frothingham, of Ann Arbor, his subject being "Surgery as an Art and a Science." In a scholarly manner he traced the progress of surgery from the early days when it was consigned to the hands of barbers up to the present time, showing its mighty advances. Rome had gone 500 years without surgery, and then assigned the work to slaves and aliens. Now surgeons occupy the front rank as regards science and learning. With the advance in scientific surgery, operations were much fewer and while in early days fatalities were frequent, the reverse was now the case. The doctor's address was listened to with great nterest, and frequently applauded.

At the close of the exercises the members, at the residence of Dr. T. A. McGraw, were tendered a reception.

SECOND DAY.

The following officers of the association were elected:

President—Dr. S. S. French, Battle Creek.

First Vice-President—Dr. Charles N. Lewis, Jackson.

Second Vice-President—Dr. E. B. Ward, Laingsburg.

Third Vice-President—Dr. Simeon Belknap, Niles.

Secretary—Dr. George Duffield, Detroit.

Treasurer—Dr. H. B. Hemenway, Kalamazoo.

Members of the Judicial Council—Drs. William Brodie, Detroit; F. K. Owen, Ypsilanti, and J. H. Bennett, Coldwater.

The society will meet at Kalamazoo, in 1889.

---

### NATIONAL ASSOCIATION OF RAILWAY SURGEONS.

The committee for making arrangements for a national meeting of railway surgeons met in the city of Fort Wayne, Indiana, on the 15th of May, 1888, and decided to hold the preliminary meeting in Chicago on the 28th of June at 9.30 a. m. Accordingly, the Association convened on the above day, and was called to order by the chairman of the committee, Dr. C. B. Stemen, of Fort Wayne, who briefly stated that the meeting was for permanent organization. The objects of the Association were to bring the surgeons of the different railroads together annually to develop this special and rapidly-growing branch of surgery; to report cases coming under their observation; to relate their experiences; to exchange views, and to discuss the best methods in the treatment of railway accidents and injuries. The meeting was large and enthusiastic, many distinguished railroad surgeons of the United States being present. Sixty-three railroads were represented. The Association starts out with a membership of six hundred and promises to be an active organization.

The morning session was devoted principally to the election of officers.

In the afternoon the following papers were read: Report of two cases of Successful Triple Amputation for Railway Injuries, by Dr. J. B. Luckie, Birmingham, Alabama.

Synchronous Amputation, by Dr. N. C. Lynd, New York.

Report of a case of Simultaneous Triple Amputation with Recovery, by Dr. A. J. Banker, Columbus, Indiana.

First Care and Treatment of Railway Injuries, by Dr. H. H. Middlecamp, Warrenton, Missouri.

Relations of Railway Surgeons and Members of the Bar, Dr. La Grange Severance, Huntington, Indiana.

External Wounds Communicating with Fractures of Bones, by Dr. J. R. Williams, White Pigeon, Michigan.

Report of a Case, by Dr. William C. Caldwell, Fremont, Ohio.

Treatment of Compound Fractures, by Dr. W. L. Buckner, Youngstown, Ohio.

Brakemen's Injured Fingers, by Dr. J. A. Jackson, Madison, Wisconsin.

Fracture of the Spine, with reports of two cases, by Dr. W. C. Henry, Aurora, Indiana.

A Few Points in Railway Surgery, by Dr. W. B. Outten, St. Louis, Missouri.

Violent Injuries of the Pelvic Bones and Pelvic Viscera, by Dr. W. A. McCandless, St. Louis, Missouri.

Dr. Outten said that out of 103 railway injuries caused by the passage of locomotives and cars over the parts attended by him, involving the superior and inferior extremities, excluding injuries of the hands or feet, more than one-third died from shock, and one-tenth died from shock with out operative interference,

Dr. J. B. Murdock, of Pittsburgh, Pennsylvania, said he had been deeply interested in Dr. Outten's paper, and in discussing railway injuries the important question is whether surgeons should adopt primary amputation, or resort to secondary amputation. He maintained that intermediate amputation was unsafe and dangerous. Military and railway surgeons know that the mortality in primary amputation is much less than in secondary amputation. After the patient had sufficiently reacted from the shock, it was his rule to perform amputation, and the sooner the better, and the greater were the chances for recovery of the patient after the operation.

Dr. H. J. Bennet, of Ohio, had practiced his profession for twenty-five years, and had treated a large number of railway accidents and injuries, but had never used carbolic acid, bichloride of mercury, nor even a drainage-tube, for in so doing he considered he added to the wound a material that would produce inflammation. He used nothing but pure lint, a plain, dry roller, etc.

Dr. C. B. Stemen, of Fort Wayne, entered his protest against those who are opposed to antiseptic surgery. Sir Joseph Lister in his teachings has been the greatest benefactor of the present age in advocating antisepsis. Although Lawson Tait, and Bantock may directly ignore it, yet a visit to their operating rooms will convince any physician that they practice it. A person has to subscribe to an oath that he has not been in a dissecting room or attending a person with an infectious disease prior to being admitted to witness their operations.

Officers for 1889.

President.—Dr. J. W. Jackson, Kansas City, Missouri.

First Vice-President.—Dr. J. H. Murphy, St. Paul, Minnesota.

Second Vice-President.—Dr. J. B. Murdock, Pittsburgh, Pennslyvania.

Third Vice-President. — Dr. A. W. Ridenour, Massillon, Ohio.

Fourth Vice-President. — Dr. B. L. Hovey, Rochester, New York.

Permanent Secretary.—Dr. C. B. Stemen, Fort Wayne, Indiana.

Assistant Secretary.—Dr. J. H. Tressel, Alliance, Ohio.

Corresponding Secretary.—Dr. E. R. Lewis, Kansas City, Missouri.

Treasurer.—Dr. J. Harvey Reed, Mansfield, Ohio.

Next place of meeting, St. Louis, Missouri.

